# Assessment of attitudes of patients with psychiatric disorders regarding electroconvulsive therapy as a treatment option

**DOI:** 10.12669/pjms.36.3.1637

**Published:** 2020

**Authors:** Ghaazaan Khan, Zahid Nazar, Mian Mukhtiar Ul Haq, Mian Iftikhar Hussain

**Affiliations:** 1 Ghaazaan Khan, Consultant Psychiatrists, Iftikhar Psychiatric Hospital, Peshawar, Pakistan; 2 Zahid Nazar, Department of Psychiatry, Lady Reading Hospital, MTI, Peshawar, Pakistan; 3 Mian Mukhtiar Ul Haq, Department of Psychiatry, Lady Reading Hospital, MTI, Peshawar, Pakistan; 4 Mian Iftikhar Hussain, Consultant Psychiatrists, Iftikhar Psychiatric Hospital, Peshawar, Pakistan

**Keywords:** Attitude, Electroconvulsive therapy

## Abstract

**Objective:**

To assess the attitudes of psychiatric patients towards electroconvulsive therapy (ECT) as a treatment modality.

**Methods:**

This descriptive cross-sectional study was conducted from 1^st^ January, 2017 to 15^th^ April, 2018 in Department of Psychiatry, MTI, Lady Reading Hospital, Peshawar. It comprised of total 154 patients, having previous experience with electroconvulsive therapy (ECT) who were selected through a non-probability consecutive sampling. Their attitude was assessed by their responses to 15 questions on a Likert Scale, each question scoring 01-05 with a summed up cut-off score of 45 points. Score over 45 points is considered positive and below 45 as negative while those scoring exactly 45 points were considered as having Ambivalent attitude towards ECT.

**Results:**

Of all, 73% patients revealed positive and 27% negative attitude towards ECT. Mean age of the sample was 35 years. Out of all patients, 67.5% were males & 32.5% females, 73% were married & 27% unmarried, 47% were illiterate & 53% variably educated, 43% were employed while 57% were unemployed.

**Conclusion:**

A significant majority of the patients accepted ECT as an effective treatment modality. However, to make the procedure more acceptable, it may be made more effective and safe to the expectations of the patients and medical professionals for better outcomes.

## INTRODUCTION

Mental disorders are being recognized as global development issues in recent years.^1^ Mental illnesses account for about 13% of global disease burden and by 2030, major depression will likely be the largest global disease burden.^2^ Currently various scientific methods are available to treat mental disorders including psychotherapy, pharmacotherapy and neuromodulatory interventions, the last one incorporating ECT.^3^

In spite of the fruitful use of ECT since 1938^4^ and its positive contribution to treat conditions like affective disorders, psychosis and catatonia, the method has been subjected to divergent opinions among the practitioners, the public, the patients and even the politicians, making it a controversial and stigmatized procedure since its inception.^5^,^6^ The negative public opinion has even led to the banning of ECT in some countries like Italy and Slovenia.^7^,^8^ Due to the negative perception portrayed regarding ECT, this otherwise highly useful modality has remained underutilized.^9^

In Khyber Pakhtunkhwa, Pakistan, ECT is an important treatment option for many psychiatric disorders and the procedure is widely practiced to a significant extent. Therefore, there is genuine need to study the attitudes of the patients with psychiatric illnesses towards ECT and the present study has been designed in this perspective.

## METHODS

A total of 154 patients fulfilling the inclusion criteria were included. The study was conducted from 1^st^ January 2017 to 15^th^ April 2018. Patients were recruited from the psychiatric outpatient and in-patient facilities of Lady Reading Hospital using non-probability consecutive sampling method. The study was conducted after taking permission from Hospital Ethical Committee (Ref No. 08/MA, dated January 31, 2018). Patients’ confidentiality was ensured throughout the study. Each patient was explained the purpose of study and his/her consent taken verbally to be part of study. A questionnaire was used to assess attitude of each patient containing 15 questions. Each individual question had a score range of 01-05 points with a score ranging from 15 to 75 points with a cut off score of 45 points. Patients scoring above 45 were considered as having positive attitude, those scoring below 45 as having negative attitude while patients scoring exactly 45 were considered as having ambivalent attitude towards ECT.

### Inclusion criteria:

1. Patients who had previous experience with electroconvulsive therapy.
2. Patients 18-60 years of age.
3. Both male and female patients.
4. Patients who could understand Pashtu, Urdu or English.

### Exclusion criteria:

1. Those who were grossly psychotic or acutely manic with no insight.
2. Those under the influence of or being treated for substance abuse.

### Data analysis:

The data gathered was analyzed using the Statistical Program for Social Sciences (SPSS), version 20. For the variables of continuous type like age, the Mean with ±S.D was calculated. For the variables of categorical type like gender, marital status, educational status, status of employment and attitude towards ECT, frequencies and percentages were calculated. Attitude was stratified among age, gender, education, employment and marital status to see any significant correlation between these variables. Post stratification chi-square test was applied to determine the effect modification. P-value less than or equal to 0.05 was taken as significant. The results are presented in the form of tables.

## RESULTS

The total number of participants was 154(n=154), all patients recruited from Psychiatry Department, Lady Reading Hospital, Peshawar. The mean age of the sample was 34.94 (S.D± 9.271). Minimum age was 18 and maximum 60 years. The various age ranges are given in Table-I. 104(67.5%) patients were males and 50(32.5%) patients were females. 113(73.4%) patients were married and 41(26.6%) patients were unmarried. As regards education, 72(46.8%) patients were illiterate, 49(31.8%) patients were educated up to matric and 33(21.4%) patients were educated above matric. 66(42.8%) patients were employed and 88 (57.2%) patients were unemployed. Out of 50 female patients only 02(1.3%) were employed while out of 104 male patients 64(41.5%) were employed while 40(26%) were unemployed. The thematic finding was that out of the participants, 112(72.7%) patients revealed positive and 42(27.3%) patient’s revealed negative attitude towards ECT. The attitude shown by various patients in different age groups are tabulated (Table-II). Out of total 104 male patients, 75(72.1%) showed positive attitude while 29(27.9%) showed negative attitude towards ECT; out of total 50 female patients 37(74%) had positive attitude while 13(26%) had negative attitude about ECT. 51(77.3%) out of 66 employed patients had positive attitude while 15(22.7%) had negative attitude regarding ECT; Among unemployed patients, 61(69.3%) had positive attitude while 27(30.7%) had negative attitude about ECT. Table-III. Among the various sociodemographic factors, only marital status has statistically significant correlation with attitude towards ECT.Table-IV.

**Table-I:** Age range of the patients.

Age Range	Frequency	Percentage
18-30 years	56	36.36
31-40 years	66	42.86
41-50 years	20	12.98
51-60 years	12	07.80

Total	154	100

**Table-II:** Attitude according to age range.

Age Range	Positive Attitude		Negative Attitude		Both

	Frequency	Percentage	Frequency	Percentage	
18-30 years	38	67.9	18	32.1	56
31-40 years	49	74.2	17	25.8	66
41-50 years	16	80	04	20	20
51-60 years	09	75	03	25	12

Total	112	72.7	42	27.3	154

p-value as per Pearson Chi-Square Test comes out to be 0.727 which is not significant.

**Table-III:** Attitude according to education.

Category	Positive Attitude		Negative Attitude		All

	Frequency	Percentage	Frequency	Percentage	
Illiterate	51	70.8	21	29.2	72
Up to Matric	33	67.3	16	32.7	49
Above Matric	28	84.8	05	15.2	33

Total	112	72.7	42	27.3	154

p-value as per Pearson Chi-Square Test comes out to be 0.193 which is not significant.

**Table-IV:** Attitude according to marital status.

Category	Positive Attitude		Negative Attitude		All

	Frequency	Percentage	Frequency	Percentage	
Married	89	78.8	24	21.2	113
Unmarried	23	56.1	18	43.9	41

Total	112	72.7	42	27.3	154

p-value as per Pearson Chi-Square Test comes out to be 0.005 which is significant.

## DISCUSSION

The thematic finding of this study has been that 112/154 patients, i.e. 72.7%, had positive attitude towards ECT whereas 42/154, i.e. 27.3%, had negative attitude towards the procedure. No ambivalent attitude has been recorded in our study as recorded in some previous studies.^10^

Adhikari SR has referred to various studies depicting that in 53-98, average, 70% of patients undergoing ECT, the procedure was perceived to be clinically beneficial.^11^ In our study, patients’ attitude towards ECT has also been in accordance with these studies.

In this study, 27.3% of the patients had negative attitude towards ECT which is in agreement with some other studies conducted in Pakistan like Ul-Haq et al. from Rawalpindi which revealed 62.6% satisfaction and 37.4% dissatisfaction with ECT^10^ which is comparable to our study with a bit higher negativity than our study.

The mean age and SD (34.94, S.D± 9.271) of our study is in the range of some other studies from Pakistan. One such local study revealed a mean age of 37.95 (S.D±12.55) and a range of 18 to 63 year.^10^ Our mean age is also comparable to some other Asian studies.^12^

The gender percentage (67.5% male, 32.5% female) of our study is nearly similar to a study conducted in Indian Kashmir where male gender comprised 64.5% and female gender comprised 35.5% of the sample.^13^ The lower percentage of female patients (32.5%) as compared to higher percentage of male patients (67.5%) in our study might be a manifestation of reduced accessibility of female gender to health care in developing countries.^14^

In our study, sociodemographic factors, excluding marital status, like age, gender, level of education and employment status had no statistical significance on the attitude of the patients towards ECT. Such findings are mostly comparable to those observed by Grover S, Chakrabarti S, Avasthi A and some others.^15^

While correlating marital status and attitude towards ECT, the p-value as per Pearson Chi-Square Test comes out to be 0.005 which is significant, meaning that marital status has a bearing on the attitude towards ECT. In married patients, 78.8% had positive attitude towards ECT. Such finding is explainable on the facts that on the one hand, as compared to the unmarried people, married people are likely to have lower risk or severity of depression^16^ and on the other hand, to be single is a poor prognostic factor in schizophrenia.^17^ A higher percentage of married participants (73.4%) in our study could be due to the trend of early marriages in Pakistan.^18^,^19^

Although there is no significant correlation between the educational level of the patients and attitude of our patients towards ECT, 72 patients (46.8%) were illiterate. This finding is consistent with the general literacy rate of Khyber Pakhtunkhwa, being described as 50%.^20^

In this study, 66(42.9%) of the patients were employed while 88(57.2%) were unemployed. Out of 50 female patients in our study, only 2(1.3%) were employed. Thus the employment status of our study is a bit lower than the employment status of the previous study by ul Haq I et al. wherein 83(50.9%) of the patients were employed and 80(49.1%) were unemployed.^10^ The low employment status of female subjects in our study is in concordance with the International Labour Organization report which has depicted gender inequality in labour force contribution rates in Pakistan where male employment approaches 80% in comparison to female employment rate of below 20%.^21^

It is suggested that media should be used to create awareness about early recognition of mental disorders and highlight the fact that ECT is an effective mode of treatment in carefully selected patients.

### Authors’ contribution:

**GK** conceived the idea, designed and executed the study, did final edits on manuscript and is responsible for integrity of research. **ZN** supervised and reviewed the study and did statistical analysis. **MMUH** assisted in preparation and scoring of Likert Scale and wrote methodology section. **MIH** assisted in preparation of manuscript.
